# Biological Potentials and Phytochemical Constituents of Raw and Roasted *Nigella arvensis* and *Nigella sativa*

**DOI:** 10.3390/molecules27020550

**Published:** 2022-01-16

**Authors:** Hussah Abdullah Alshwyeh, Sahar Khamees Aldosary, Muna Abdulsalam Ilowefah, Raheem Shahzad, Adeeb Shehzad, Saqib Bilal, In-Jung Lee, Jannah Ahmed Al Mater, Fatima Najf Al-Shakhoari, Waad Abdulrahman Alqahtani, Nurkhalida Kamal, Ahmed Mediani

**Affiliations:** 1 Department of Biology, College of Science, Imam Abdulrahman Bin Faisal University, Dammam 31441-1982, Saudi Arabia; skdosary@iau.edu.sa (S.K.A.); adeeb.shahzad@gmail.com (J.A.A.M.); saqib043@yahoo.com (F.N.A.-S.); ijlee@knu.ac.kr (W.A.A.); 2 Department of Food Technology, Faculty of Engineering and Technology, Sabha University, Sabha, Libya; stardream-17@hotmail.com; 3 Department of Horticulture, The University of Haripur, Haripur 22620, Khyber Pakhtunkhwa, Pakistan; shakhoarifn@hotmail.com; 4 Department of Biomedical Sciences, School of Mechanical and Manufacturing Engineering (SMME), National University of Sciences and Technology (NUST), Bolan Road, H-12, Islamabad 44000, Pakistan; waadalqahtani97@gmail.com; 5 Natural and Medical Sciences Research Center, University of Nizwa, Nizwa 616, Oman; mona.milad2005@gmail.com; 6 School of Applied Biosciences, Kyungpook National University, Daegu 41566, Korea; raheem.shahzad@uoh.edu.pk; 7 Institute of Systems Biology, Universiti Kebangsaan Malaysia (UKM), Bangi 43600, Selangor, Malaysia; nurkhalida.kamal@ukm.edu.my

**Keywords:** *Nigella arvensis*, *Nigella sativa*, antimicrobial activity, MTT, methanol extract, GC–MS

## Abstract

*Nigella* species are widely used to cure various ailments. Their health benefits, particularly from the seed oils, could be attributed to the presence of a variety of bioactive components. Roasting is a critical process that has historically been used to facilitate oil extraction and enhance flavor; it may also alter the chemical composition and biological properties of the *Nigella* seed. The aim of this study was to investigate the effect of the roasting process on the composition of the bioactive components and the biological activities of *Nigella arvensis* and *Nigella sativa* seed extracts. Our preliminary study showed that seeds roasted at 50 °C exhibited potent antimicrobial activities; therefore, this temperature was selected for roasting *Nigella* seeds. For extraction, raw and roasted seed samples were macerated in methanol. The antimicrobial activities against *Streptococcus agalactiae, Streptococcus epidermidis, Streptococcus pyogenes, Candida albicans, Escherichia coli, Enterobacter aerogenes, Klebsiella pneumoniae,* and *Klebsiella oxytoca* were determined by measuring the diameter of the zone of inhibition. The cell viability of extracts was tested in a colon carcinoma cell line, HCT-116, by using a microculture tetrazolium technique (MTT) assay. Amino acids were extracted and quantified using an automatic amino acid analyzer. Then, gas chromatography–mass spectrometry (GC–MS) analysis was performed to identify the chemical constituents and fatty acids. As a result, the extracts of raw and roasted seeds in both *Nigella* species showed strong inhibition against *Klebsiella oxytoca,* and the raw seed extract of *N.* *arvensis* demonstrated moderate inhibition against *S. pyogenes*. The findings of the MTT assay indicated that all the extracts significantly decreased cancer cell viability. Moreover, *N. sativa* species possessed higher contents of the measured amino acids, except tyrosine, cystine, and methionine. The GC–MS analysis of extracts showed the presence of 22 and 13 compounds in raw and roasted *N. arvensis*, respectively, and 9 and 11 compounds in raw and roasted *N. sativa*, respectively. However, heat treatment decreased the detectable components to 13 compounds in roasted *N. arvensis* and increased them in roasted *N. sativa*. These findings indicate that *N. arvensis* and *N. sativa* could be potential sources of anticancer and antimicrobials, where the bioactive compounds play a pivotal role as functional components.

## 1. Introduction

Some plants and their products are a major source of drugs for much of the world’s population [1]. The World Health Organization (WHO) estimates that 80% of those in underdeveloped countries rely on traditional medicine for basic health care, and around 85% on herbal extracts [2]. Plants have inspired new therapeutic molecules since herbal remedies have significantly contributed to human health and well-being [3]. Medicinal plants are the richest bio-resource in conventional medicinal systems, nutraceuticals, food additives, contemporary medications, medicinal products, folk remedies, and synthetic chemical entities. The WHO has proposed that a wide range of medicines should ideally be obtained from medicinal plants [2].

The *Nigella* genus, which belongs to the Ranunculaceae family, is widespread throughout Europe, North Africa, and Asia. In folk medicine, many species of *Nigella* were found to have a significant role. *Nigella sativa* (black seeds) is one of the main plants that is traditionally utilized in folk medicine to cure several diseases, such as stomachic, diuretic, liver tonic, and diaphoretic diseases in different cultures, including Arab and Chinese cultures [4,5]. Furthermore, *Nigella arvensis* is used locally on cakes and bread as a flavoring agent, as well as a remedy for stomach pains, ulcers, and for diuretic effects [6]. It was reported that *N. sativa* exhibited antioxidant, anti-inflammatory, antimicrobial, anti-carcinogenic and could enhance learning and memory activities [4,6,7,8,9,10,11,12], while the seeds of *N. arvensis* were reported to have antimicrobial, antiallergic, antiviral, and anti-inflammatory effects (for worm infestation) [13]. The benefits of these seeds could be related to their content of many different phytochemical constituents [6]. The phytochemicals present in the black seeds include a yellowish, volatile oil (0.5–1.6%), and a fixed oil (35.6–41.6%) that possess unsaturated fatty acids (arachidonic, eicosadienoic, linoleic, and linolenic), campesterol, stigmasterol, β-sitosterol, α-spinasterol, (+)-limonene, and (+)-citronellol [7,14]. In addition to several amino acids, fibers, minerals, and vitamins were found in this seed, as well as other chemicals, including niacin, ascorbic acid, thiamine, pyridoxine, folic acid, and phenolics [5]. These molecules can be sensitive to certain processing methods.

Roasting is an important process that is traditionally used to facilitate oil extraction and enhance flavor. Accordingly, it is important to study the effects of heat treatments on black seeds in terms of the biological and chemical properties of the seeds and the extract. Additionally, the roasting process might influence the components of black seeds, such as fatty acid compounds, amino acids, vitamins, and phenolics, which would affect their antioxidant, anticancer, and antimicrobial activities. *N. arvensis* is a minor crop that is used as a substitute for *N. sativa* in some areas. Only these two species can be found throughout the Middle East, and *N. sativa* is the most globally studied in the genus. So far, there is no study that reports the comparison of cytotoxicity and antimicrobial activities between these two species. Therefore, this study aimed to investigate the anticancer and antimicrobial activities of raw and roasted *N. arvensis* and *N. sativa*. The profiling of amino acids and the identification of the phytochemicals were also investigated using GC–MS of these plants based on the roasting temperature. The obtained results could help to show the effect of roasting on the biological characteristics of the seeds and their products.

## 2. Materials and Methods

### 2.1. Materials

The instruments and the materials that were used in this study include a GC–MS Agilent 7890 A (Agilent Technologies Inc., Wilmington, DE, USA). The black seeds were obtained from a local market in the middle of Saudi Arabia and then kept in chilled, airtight containers to prevent the effects of humidity until the time of use. Pathogenic bacterial strains *Klebsiella pneumoniae, Enterobacter aerogenes, Escherichia coli, Klebsiella oxytoca, Streptococcus agalactiae, Streptococcus epidermidis, Streptococcus pyogenes,* and *Candida albicans* were obtained from the microbiology laboratory of King Fahad Hospital in Khobar, Saudi Arabia. All the other chemicals and solvents used were analytical grade.

### 2.2. Sample Preparation and Extraction

The seeds were cleaned, washed with water, air-dried for 7 days, and kept at 0 °C. After that, the seeds were roasted at different temperatures (50, 80, 100, 150, and 200 °C) and then ground into powder using a grinder (KHIND BL1012). Based on the preliminary results of antimicrobial activity, the seeds roasted at 50 °C for both species were further selected for the analysis and compared to the raw seeds. 50% Methanol was used to macerate the raw and roasted black seed powder for 24 h. The extract was separated using Whatman No. 1 filter paper. The extract was then filtered again using sodium sulfate to eliminate any remaining moisture [8]. The obtained filtrates were used for additional phytochemical analyses and biological activity.

### 2.3. Antimicrobial Activity Determination

Pathogenic bacterial strains such as *E. coli, K. pneumoniae, K. oxytoca, E. aerogenes, S. agalactiae, S. epidermidis, S. pyogenes*, and *C. albicans* were used to evaluate antimicrobial activity of the raw and roasted seeds. Antimicrobial activity was measured using agar disc diffusion method [15]. Petri dishes with 20 mL of nutrient agar were prepared, incubated with 1 × 10^6^ cell/mL, and 100 µL of a 24 h broth culture of tested pathogens. Each of discs (6 mm in diameter) was made and filled with 100 µL of raw and roasted *N. arvensis* and *N. sativa* extracts. The plates were incubated at 37 °C for 24 h. The diameter of the inhibitory zone was measured in mm after incubation.

### 2.4. Cell Viability Assay

The cell viability assay was carried out according to method described by Shahzad et al. [16]. Briefly, the HCT-116 cell lines were cultured at 2 × 10^5^ cell density in each well of 96-well plates with different concentrations of extracts (1, 5, 10, 20, 30, and 50%) and incubated at 37 °C. After incubation, the medium was replaced with 20 μL of MTT solution (5 mg mL^−1^) and incubated (37 °C for 2 h). After incubation, MTT solution was replaced with 200 μL of DMSO. The plates were gently shaken for 1 min before the optical density was measured at 540 nm using a 96-well microplate reader. The same procedure was followed for curcumin as positive standard. The cell viability was determined using the equation below:

Cell viability (%) = (OD of treated sample/OD of untreated sample) × 100

where OD is the absorbance of cells.

### 2.5. Amino Acid Profile Determination

Amino acids were extracted and quantified according to the protocol described by Shahzad et al. [17], using automatic amino acid analyzer (Hitachi Japan, L-8900).

### 2.6. Gas Chromatography–Mass Spectrometry (GC–MS) Analysis

*N. arvensis* and *N. sativa* were extracted following the method of maceration, which was completed using absolute methanol with shaking at room temperature [18]. Maceration is a method that is suitable for compounds that are sensitive to heating [19,20]. The mechanism of this process involves mass transfer of substance into the solvent, where the movement begins to occur at the interface layer and then diffuses into the solvent [18].

The samples were first derivatized in preparation for GC–MS analysis [18]. In 2 mL centrifuge tubes, the sample extract (25 mg) was mixed with 50 mL pyridine and sonicated for 10 min at 30 °C. After adding 100 mL of methoxyamine HCl (20 mg/mL in pyridine), the sample solutions were vortexed. In a shaking incubator, the mixture was incubated for 2 h at 60 °C (VorTemp 56, Labnet International, Inc., Woodbridge, NJ, USA). After that, the tube was incubated for 30 min at 60 °C with 300 mL of *N*-Methyl-*N*-(trimethylsilyl)trifluoroacetamide (MSTFA). Finally, the sample solutions were filtered, covered with aluminum foil, and allowed to stand at room temperature overnight. The seed extracts were analyzed using GC–MS in an Agilent 7890 A instrument with a controlled computer at 70 eV, using a micro syringe. One microliter of the 50% methanol extract of raw and roasted *N. arvensis* and *N. sativa* was injected into the GC–MS and scanned for 45 min. The mass/charge (*m/z*) ratio was calibrated using the acquired graph, which was dubbed the mass spectrum graph that is a molecule’s fingerprint [21]. The temperature of the oven, the gas flow rate, and the electron gun were all programmed before analyzing the extract using GC–MS. The oven temperature was retained at 100 °C. Helium gas was utilized as both a carrier and an eluent. The helium flow rate was set to 1 mL/min. The energy of the electron gun of mass detector, which liberates electrons, was around 70 eV. Elite 1 (100% dimethyl poly siloxane) is the column used for component separation. The compounds in the extracts were identified by comparing their retention time and mass spectra fragmentation patterns to those in the library, as well as those in publication. The spectra of compounds were compared to those of the NIST/EPA/NIH mass spectral and Wiley libraries [22,23].

## 3. Results and Discussion

### 3.1. Antimicrobial Activity

Based on a preliminary study, antimicrobial examinations were only conducted on the extracts of roasted seeds at 50 °C and compared to raw seeds. Table 1 represents the antimicrobial activity of raw and roasted *N. arvensis* and *N. sativa* extracts against eight types of pathogenic bacteria and candida species. The results generally revealed different effects ranging between low and moderate, where *Streptococcus pyogenes* and *Klebsiella oxytoca* were more sensitive to both types of *Nigella* species, followed by *Streptococcus epidermidis*. *N. arvensis* was more effective with inhibition zones (IZ) ranging from 10 to 35 and 20 to 25 mm for raw and roasted seeds, respectively. The extract from the raw *N. arvensis* demonstrated the highest IZ with 35 mm on *S. pyogenes*. The raw seeds exhibited higher antimicrobial activity compared to roasted ones, which could possibly be due to the negative effect of heat treatment on the compounds attributed to this bioactivity. In general, *N. arvensis* extract had slightly higher antimicrobial properties than *N. sativa* extract. This could be due to the presence of more bioactive constituents.

Note that the Gram-positive tested bacteria were more sensitive and susceptible to the extracts than the negative ones, whereas *E. coli, K. pneumoniae, K. oxytoca,* and *E. aerogenes* were resistant to most of the tested extracts. *K. oxytoca,* which is considered highly susceptible to both raw extracts, showed a 30 mm IZ. These obtained results agree with Salman et al. [24], who reported that the extracts of the black seeds were effective in the inhibition of the Gram-positive bacteria in comparison to the Gram-negative bacteria, including drug-resistant bacteria. It can be suggested that the lipid layers found in the cell wall structure of Gram-negative bacteria could be a protective layer of the Gram-negative bacteria. Table 1 shows that the effects of *N. arvensis* and *N. sativa* extracts on the tested pathogens ranged between low and moderate. Their antimicrobial action could be attributed to the presence of functional components, especially thymoquinone and melanin [25,26]. Additionally, Forouzanfar et al. [27] reported that thymoquinone (TQ) is one of the most active constituents in black seeds and has different beneficial properties. Another study concluded that the antibacterial activities of *Nigella* were the weakest, but not negligible, compared to the activities of diverse extracts from propolis, *Spilanthes oleracea*, and black garlic on various micro-organisms involved in total human salivary bacteria and in oral diseases [28].

### 3.2. Cell Viability Assay

The cell lines (HCT-116) were treated with different concentrations of the extract (0, 1, 5, 10, 20, 30, and 50%) of *N. arvensis* and *N. sativa* roasted at 50 °C. This revealed that both plants can decrease cell viability (Table 2). The highest concentration (50%) of *N. sativa* and *N. arvensis* showed 77.08 (± 5.8) and 79.64% (± 6.67) viable cells, respectively. The raw *N. sativa* and *N. arvensis* recorded 82.15 (± 6.12) and 92.03% (± 3.35) viable cells, respectively. By comparing both species, *N. arvensis* exhibited a slightly higher cell viability than *N. sativa*. The raw seeds showed better cell viability compared to the roasted ones. This could possibly be due to the effect of roasting temperature.

### 3.3. Amino Acid Profile Determination

It can be observed that both types of the studied black seeds are considered a good source of the essential amino acids except methionine (Table 3). Moreover, *N. sativa* significantly (*p* < 0.05) possessed higher contents of the measured amino acids except for tyrosine, cystine, and methionine in comparison to *N*. *arvensis*. A roasting process at 50 °C significantly increased all the amino acids except methionine compared to the raw seeds. The detectable amino acids in the current study are consistent with the findings found in other studies [5], with arginine, aspartate, and glutamate recording the highest values, and methionine, the lowest. The positive effects of heat treatment could be due to deterioration of the cell wall of the seed and the release of the associated protein, which might increase the content of amino acids during extraction.

### 3.4. GC–MS Profiles of N. arvensis and N. sativa

GC–MS analysis was carried out for the methanolic extracts of raw *N. arvensis* and *N. sativa* species. Twenty-two compounds were identified from non-roasted *N. arvensis* (Figure S1a), and nine compounds were detected in non-roasted *N. sativa* (Figure S1b). Heat treatment of the studied samples at 50 °C decreased the detectable components from 15 to 13 compounds in *N. arvensis* and increased them in *N. sativa* compared to their counterparts (Figure S1c,d). All the identified compounds were mainly fatty acids and esters. Linoelaidic and 9,12-octadecadienoic acids were the most common components in all extracts of raw and roasted *N. arvensis* and *N. sativa*. Nine compounds, namely *n*-hexadecanoic acid, hexadecenoic acid ethyl ester, oleic acid, linoleic acid ethyl ester, ethyl oleate, octadecanoic acid ethyl ester, hexadecenoic acid, 2-hydroxy-1-(hydroxy-methyl) ethyl ester, and 9,12-octadecadiene-1-ol were the main distinguished constituents in non-roasted *N. arvensis*, roasted *N. arvensis,* and roasted *N. sativa*. Compounds identified in roasted *N. arvensis* and *N. sativa* were quite similar; in contrast, there were no similar single compounds found between non-roasted *N. arvensis* and *N. sativa*. Meanwhile, 11 typical phytochemicals were identified in non-roasted *N. arvensis* (Table 4), although 4 typical compounds were found in non-roasted *N. arvensis* to be different from other compounds in the other extracts. The description of the identified phytochemicals in all extracts is summarized in Table 4. A previous qualitative investigation of *N. sativa* seeds revealed the presence of sterols, triterpenes, tannins, flavonoids, cardiac glycosides, alkaloids, and saponins [8].

Fatty acids are essential components of plants and have been established as a well-known antibacterial agent [29]. Numerous fatty acids have been identified or isolated and have exhibited effective inhibitory potential against different bacteria. Of interest from the previous studies is the synergistic effects of oleic and linoleic acids’ inhibitory activities against *Staphylococcus aureus* and *Kocuria kristinae* with MIC of 0.05 mg/mL [23,30]. Meanwhile, esters of fatty acids are also food additives with strong inhibitory activity against different *Bacillus* species, namely, *Bacillus cereus, Bacillus subtilis, Bacillus megaterium,* and *Bacillus coagulans* [31].

## 4. Conclusions

The *N. arvensis* and *N. sativa* seed extract exhibited relatively moderate inhibition activities against different tested microbes and anticancer activity, with *N. arvensis* having higher activity than *N. sativa*. Both species are a good source of many bioactive compounds, such as amino acids and fatty acid compounds. The raw seeds showed better activity and preserved most compounds in both species compared to roasting at 50 °C. This study has given pertinent information on the potential antimicrobial and anticancer effects of *N. arvensis* and *N. sativa* extracts, as well as the phytochemical constituents. The findings support the practice where *N. arvensis* is a substitute for *N. sativa* in some regions of the world. The chemical components present in these species may be useful for various herbal formulations. More research is required, especially in vivo assays and determining their medicinal significance.

## Figures and Tables

**Table 1:** Antimicrobial activity of raw and roasted *N. arvensis* and *N sativa*.

Micro-Organisms	Inhibition Zone (mm)				
	Raw		Roasted		Amoxicillin (500 mg)
	*N. arvensis*	*N. sativa*	*N. arvensis*	*N. sativa*	
*Streptococcus epidermidis*	22	17	20	15	60
*Streptococcus pyogenes*	35	20	20	13	50
*Candida albicans*	20	14	ND	12	70
*Streptococcus agalactiae*	ND	ND	ND	ND	30
*Escherichia coli*	10	12	ND	ND	50
*Klebsiella pneumoniae*	10	Re	ND	ND	30
*Klebsiella oxytoca*	30	30	25	20	30
*Enterobacter aerogenes*	ND	10	ND	ND	30

ND: Not determined.

**Table 2:** Effects of raw and roasted *Nigella arvensis* (R) and *Nigella sativa* (S) at different extract concentrations (0, 1, 5, 10, 20, 30, and 50%) on HT-116 cell cancer viability.

Extract Concentration (%)	Raw R	Roasted R	Raw S	Roasted S	Curcumin
1%	104.94 ± 8.85 ^a^	101.84 ± 1.44 ^b^	97.57 ± 5.94 ^c^	98.60 ± 8.75 ^c^	94.03 ± 2.56
5%	98.08 ± 1.20 ^a^	91.81 ± 6.96 ^d^	94.99 ± 4.13 ^c^	96.24 ± 3.21 ^b^	99.34 ± 2.24
10%	99.63 ± 1.63 ^a^	98.08 ± 11.10 ^a^	91.52 ± 1.16 ^c^	93.88 ± 2.63 ^b^	92.48 ± 3.46
20%	92.70 ± 3.09 ^c^	100.59 ± 5.42 ^a^	94.47 ± 4.74 ^b^	91.15 ± 3.89 ^c^	86.50 ± 3.67
30%	93.51 ± 2.90 ^a^	87.54 ± 4.60 ^b^	83.48 ± 2.51 ^c^	85.25 ± 6.11 ^d^	88.05 ± 4.12
50%	92.04 ± 2.73 ^a^	82.15 ± 5.00 ^b^	79.65 ± 5.46 ^c^	77.08 ± 4.77 ^d^	75.00 ± 1.67

R—*Nigella arvensis*; S—*Nigella sativa*. The values are means ± standard deviation for three replicates. The values are the mean ± standard deviation for three replicates; the means with the same superscribed letter in the same row are not significantly different (*p* > 0.05).

**Table 3:** Amino acid (mg/g, dw) profile of raw and roasted *N. arvensis* (R) and *N. sativa* (S).

Treatment	Asp	Thr	Met	ILE	Ser	Glu	Leu	Tyr	Gly	Phe	Lys	Cys	Val	His	Arg	Ala	Pro
Raw R	40.20 ± 0.89 ^a^	17.73 ± 0.52 ^b^	6.86 ± 0.66 ^b^	19.15 ± 0.69 ^b^	20.10 ± 0.11 ^a^	76.61 ± 2.33 ^a^	31.27 ± 0.96 ^a^	18.12 ± 0.34 ^a^	27.91 ± 0.60 ^a^	19.69 ± 0.78 ^a^	21.73 ± 1.70 ^a^	8.26 ± 0.34 ^a^	23.65 ± 0.60 ^a^	13.7 ± 0.67 ^a^	40.13 ± 2.09 ^a^	20.21 ± 0.19 ^a^	56.10 ± 1.61 ^a^
Roasted R	46.98 ± 1.72 ^b^	21.53 ± 0.38 ^a^	8.85 ± 0.35 ^a^	22.34 ± 0.25 ^a^	24.10 ± 0.80 ^a^	86.33 ± 0.32 ^b^	40.16 ± 0.69 ^b^	20.14 ± 0.91 ^a^	32.76 ± 0.27 ^b^	23.80 ± 0.23 ^b^	28.72 ± 1.33 ^b^	11.50 ± 3.28 ^b^	26.53 ± 0.63 ^b^	17.2 ± 0.78 ^b^	47.75 ± 0.54 ^b^	24.03 ± 0.04 ^b^	62.04 ± 5.86 ^b^
Raw S	72.36 ± 1.35 ^c^	28.45 ± 0.69 ^b^	2.05 ± 0.06 ^a^	31.36 ± 0.94 ^b^	36.59 ± 1.40 ^b^	104.74 ± 0.36 ^c^	71.79 ± 1.33 ^c^	19.18 ± 1.19 ^a^	49.58 ± 1.14 ^c^	38.62 ± 0.17 ^c^	50.45 ± 0.54 ^c^	5.96 ± 0.94 ^c^	31.25 ± 0.47 ^c^	19.5 ± 1.06 ^c^	70.15 ± 0.45 ^c^	37.71 ± 1.10 ^c^	95.59 ± 1.44 ^c^
Roasted S	79.78 ± 0.86 ^d^	32.59 ± 0.42 ^a^	2.03 ± 0.06 ^a^	34.10 ± 0.41 ^a^	42.54 ± 1.13 ^c^	107.95 ± 1.66 ^c^	79.57 ± 1.25 ^d^	21.74 ± 0.61 ^a^	55.01 ± 0.28 ^d^	44.45 ± 1.56 ^d^	55.83 ± 1.02 ^d^	8.29 ± 0.57 ^a^	36.83 ± 1.23 ^d^	23.0 ± 0.21 ^d^	75.99 ± 0.86 ^d^	44.04 ± 1.10 ^d^	106.16 ± 1.80 ^d^

Asp—Aspartic acid; Thr—Threonine; Met—Methionine; ILE—Isoleucine; Ser—Serine; Glu—Glutamic acid; Leu—Leucine; Tyr—Tyrosine; Gly—Glycine; Phe—Phenylalanine, Lys—Lysine; Cys—Cysteine; Val—Valine; His—Histidine; Arg—Arginine; Ala—Alanine; Pro—Proline. The values are the mean ± standard deviation for three replicates; the means with the same superscribed letter in the same column are not significantly different at *p* > 0.05.

**Table 4:** GC–MS Analysis of the phytochemicals of raw and roasted *N. arvensis* and *N. sativa*.

Compound’s Name	Area % and Retention Times of Identified Constituents in Each Sample			
	Raw *N. arvensis* (RT)	Roasted *N. arvensis* (RT)	Raw *N. sativa* (RT)	Roasted *N. sativa* (RT)
*N*-1-(4-hydroxybutyl)-*N*-3-methylguanidine acetate	0.58 (19.82)			
Tetradecanoic acid	0.09 (29.14)			
Tetradecanoic acid ethyl ester	0.11 (30.14)			
*n*-hexadecanoic acid	9.95 (34.49)	8.54 (34.40)		8.54(34.40)
Ethyl 9-hexadecanoate	0.36 (34.71)			
Hexadecenoic acid ethyl ester	4.12 (35.21)	2.03 (35.19)		2.03 (35.19)
9,12-octadecadienoic acid	23.79 (38.74)	27.3 (38.59)	19.37 (38.44)	27.34 (38.59)
Oleic acid	10.39 (38.84)	15.15 (38.70)		15.15 (38.70)
Linoleic acid ethyl ester	17.73 (39.20)	13.40 (39.15)		13.40 (39.15)
Ethyl oleate	8.95 (39.32)	4.54 (39.27)		4.54 (39.27)
9-octadecanoic acid ethyl ester	0.64 (39.42)			
Octadecanoic acid ethyl ester	0.82 (39.82)	0.28 (39.81)		0.28 (39.81)
9,12-octadecadienoic acid	0.36 (42.83)			
7-pentadecyne	0.27(42.98)			
9-octadecanoic acid,12-hydroxyethyl ester	0.14 (43.35)			
9,17-octadecadienal	0.69 (43.44)			
Oxacyclotricosan-2-one	0.16 (43.55)			
Hexadecenoic acid, 2-hydroxy-1-(hydroxy-methyl) ethyl ester	2.34 (46.45)	3.06 (46.44)		3.06 (46.44)
(*Z, Z*)-9,12-octadecadienoic acid,2-hydroxy-1-(hydroxy-methyl) ethyl ester	9.63 (49.896)			
9,12-octadecadiene-1-ol	7.57 (46.95)	12.52 (49.92)		12.50 (49.92)
Octadecanoic acid, 2,3-dihydroxypropylester	0.83 (50.32)			
Linoelaidic acid	0.28 (43.45)	0.28 (43.45)	10.54 (38.56)	0.28 (43.45)
13- octadecanal		1.14 (50.13)		1.14 (50.13)
5-hydroxy methyl furfural			2.92 (13.57)	
2,5-cyclohexadiene-1,4-dione			1.83 (23.80)	
Inositol			37.57 (27.04)	
2-(diethylamino)-*N*-dimethylphenyl acetamide			1.11 (32.89)	

RT—retention time.

## Data Availability

All data of this study are presented within the study.

## Supplementary Materials

The following supporting information can be downloaded, Figure S1: GC-MS chromatograms of raw *Nigella arvensis* (a), raw *Nigella sativa* (b), roasted *Nigella arvensis* (c) and roasted *Nigella sativa* (d) seed extracts.

## Sample Availability

Samples of the compounds are available from the authors.
