# Supplementary material for: Biological Potentials and Phytochemical Constituents of Raw and Roasted Nigella arvensis and Nigella sativa
Source: Molecules. 2022 Jan 16;27(2):550. doi: 10.3390/molecules27020550 (PMC8779992; doi:10.3390/molecules27020550)
Supplement: Supplementary file 1 [file molecules-27-00550-s001.zip › molecules-1529095-supplementary.pdf]

Supplementary Materials

Biological potentials and phytochemical constituents of raw and roasted *Nigella arvensis* and *Nigella sativa*

Hussah Abdullah Alshwyeh [1,*], Sahar Khamees Aldosary [1], Muna Abdulsalam Ilowefah [2], Raheem Shahzad [3], Adeeb Shehzad [4], Saqib Bilal [5], In-Jung Lee [6], Jannah Ahmed Al Mater [1], Fatima Najf Al-Shakhoari [1],
Waad Abdulrahman Alqahtani [1], Nurkhalida Kamal [7] and Ahmed Mediani [7,*]

[1] Department of Biology, College of Science, Imam Abdulrahman Bin Faisal University, Saudi Arabia; skdpsary@iau.edu.sa; stardream-17@hotmail.com; shakhoarifn@hotmail.com; waadalqahtani97@gmail.com.
[2] Department of Food Technology, Faculty of Engineering and Technology, Sabha University, Sabha, Libya; mona.milad2005@gmail.com.
[3] Department of Horticulture, The University of Haripur, Haripur, Pakistan; raheem.shahzad@uoh.edu.pk.
[4] Department of Biomedical Sciences, School of Mechanical and Manufacturing Engineering (SMME), National University of Sciences and Technology (NUST), H-12, Islamabad, Pakistan; adeeb.shahzad@gmail.com.
[5] Natural and Medical Sciences Research Center, University of Nizwa, Nizwa 616, Oman; saqib043@yahoo.com.
[6] School of Applied Biosciences, Kyungpook National University, Daegu, South Korea; ijlee@knu.ac.kr.
[7] Institute of Systems Biology, Universiti Kebangsaan Malaysia (UKM), Bangi 43600 UKM, Selangor, Malaysia; nurkhalida.kamal@ukm.edu.my.
*Correspondence: medianiahmed47@gmail.com (A.M.); haalshuyeh@iau.edu.sa

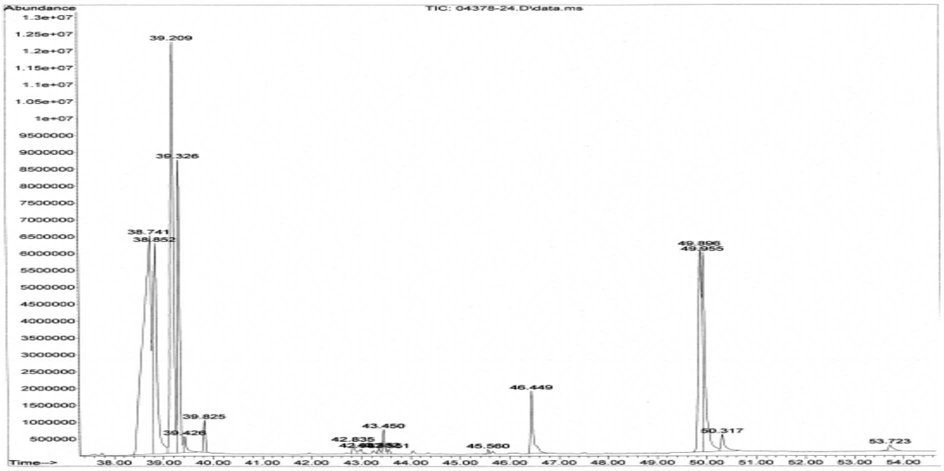
Abundance
TIC: 04378-24.D\data.ms
39.209
39.326
38.741
38.852
39.426
39.825
42.835
43.450
45.560
46.449
49.896
49.955
50.317
53.723
Time-->

a.

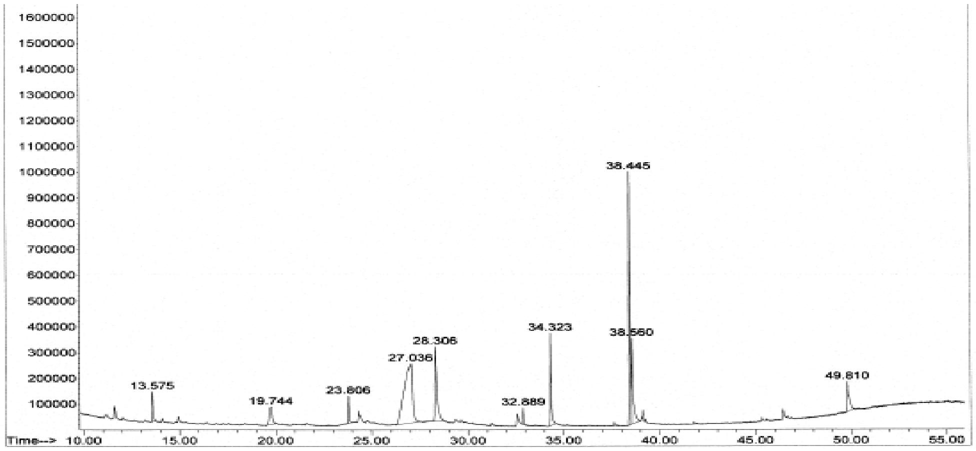
13.575
19.744
23.806
27.036
28.306
32.889
34.323
38.445
38.560
49.810
Time-->

b.

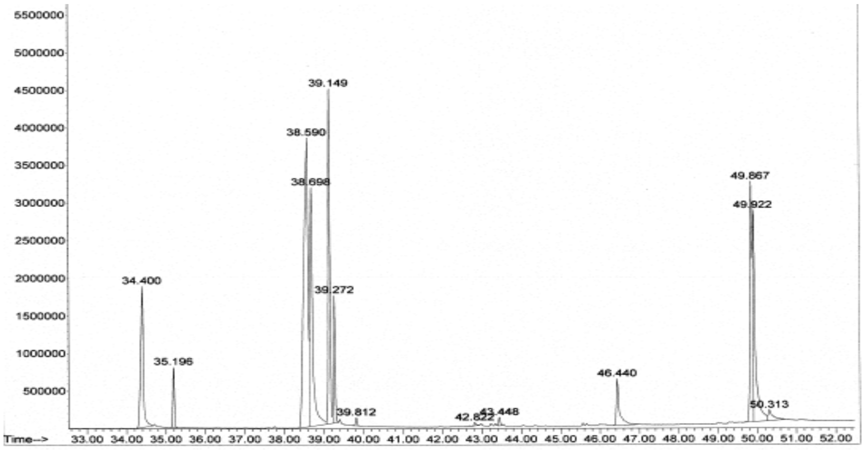
34.400
35.196
38.590
38.698
39.149
39.272
39.812
42.822
43.448
46.440
49.867
49.922
50.313
Time-->

c.

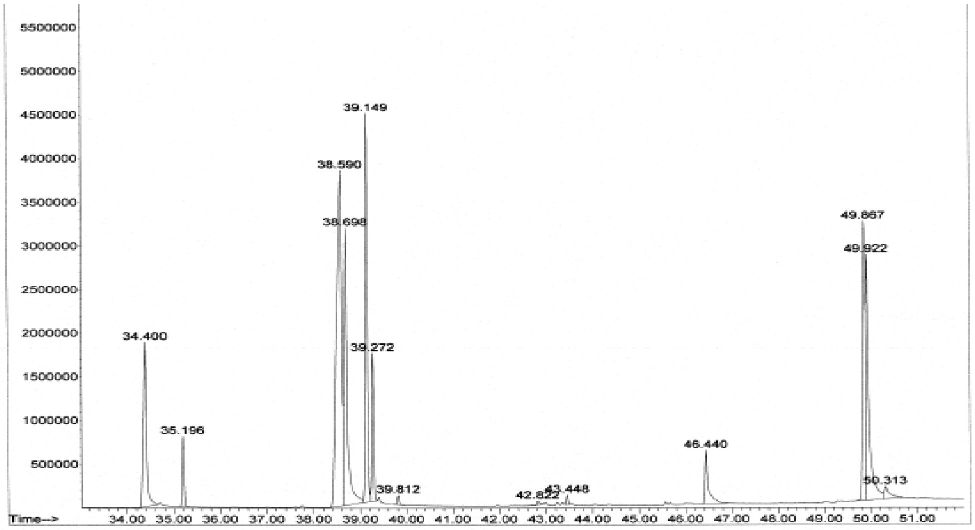

d.

Figure S1. GC-MS chromatograms of raw *Nigella arvensis* (a), raw *Nigella sativa* (b), roasted *Nigella arvensis* (c) and roasted *Nigella sativa* (d) seed extracts.
